# Clinical Characteristics of Severe COVID-19 Patients Admitted to an Intensive Care Unit in Lombardy During the Italian Pandemic

**DOI:** 10.3389/fmed.2021.582896

**Published:** 2021-03-25

**Authors:** Matteo Briguglio, Tiziano Crespi, Fabio Pino, Marco Mazzocchi, Mauro Porta, Elena De Vecchi, Giuseppe Banfi, Paolo Perazzo

**Affiliations:** ^1^IRCCS Orthopedic Institute Galeazzi, Scientific Direction, Milan, Italy; ^2^Intensive Care Unit, IRCCS Orthopedic Institute Galeazzi, Milan, Italy; ^3^Neurology Unit, IRCCS Orthopedic Institute Galeazzi, Milan, Italy; ^4^Laboratory of Clinical Chemistry and Microbiology, IRCCS Orthopedic Institute Galeazzi, Milan, Italy; ^5^Faculty of Medicine and Surgery, Vita-Salute San Raffaele University, Milan, Italy

**Keywords:** SARS-CoV-2, COVID-19, infection, intensive care, intubation, systemic inflammatory response syndrome, sepsis, anesthesia

## Abstract

Italy was one of the worst affected European countries during the severe acute respiratory syndrome coronavirus 2 (SARS-CoV-2) pandemic. More than 50% of Italian cases occurred in the northern region of Lombardy, where the saturation of health services between March and April 2020 forced hospitals to allocate patients according to available resources. Eighteen severe coronavirus disease 2019 (COVID-19) patients were admitted to our hospital needing intensive support. Given the disease fatality, we investigated the patients' characteristics to identify mortality predictors. We counted seven deaths from multiple organ failure, two from septic shock, and two from collapsed lungs. The maximum case fatality was observed in patients who contracted SARS-CoV-2 in hospitals. The fatal outcome was associated with the following baseline characteristics: polymorbidity (OR 2.519, *p* = 0.048), low body mass index (OR 2.288, *p* = 0.031), low hemoglobin (OR 3.012, *p* = 0.046), and antithrombin III (OR 1.172, *p* = 0.048), along with a worsening of PaO_2_/FiO_2_ ratio in the first 72 h after admission (OR 1.067, *p* = 0.031). The occurrence of co-infections during hospitalization was associated with a longer need for intensive care (B = 4.511, *p* = 0.001). More information is needed to inform intensive care for patients with severe COVID-19, but our findings would certainly contribute to shed some light on this unpredictable and multifaceted disease.

## Introduction

The severe acute respiratory syndrome coronavirus 2 (SARS-CoV-2) is a respiratory virus that primarily affects the lungs of the human host and that causes, in susceptible individuals, an unrestrained response of the immune system, respiratory failure, cardiovascular system damage, neuropsychiatric manifestations, and multiple organ injuries (1–4). Since the first outbreak testimony in China at the end of the year 2019, the apparent disease termed coronavirus disease 2019 (COVID-19) had a large-scale spread within a few months. Among the European countries, Italy was the first to confront the worst infection situation, and it has been for many weeks the nation most affected, with hundreds of deaths being testified every day. About 60,000 total deaths have been presently confirmed (WHO weekly epidemiological update, 8 December 2020) and over half of all losses having occurred in the northern region of Lombardy, in many respects considered the epicenter of the Italian economic and industrial activity. The number of new cases has been progressively decreasing since May 2020, but at the peak of the emergency scenario in March–April 2020, all the major hospitals in northern Italy were forced to cope with this infection, rapidly corroborating the saturation of the health services (5). Along with the consistent traumatic injuries, patients with severe COVID-19 overflowing from the surrounding clinics were transferred to our hospital in Lombardy (6). In critically ill COVID-19 patients, it is a fact that respiration support saves lives, but reports from the United States, Canada, United Kingdom, and China have observed survival rates ranging from 15 to 97% (7–11), with the dissimilar signs and biochemical fluctuations possibly depending upon the variety of environmental factors and ethnicity (12, 13). Given the need for better characterization of severe COVID-19 patients in our Italian context, the aim of this study is to describe albeit retrospectively the clinical and biochemical characteristics of the COVID-19 patients admitted to the intensive care unit (ICU) of our hospital.

## Materials and Methods

Patients admitted to our hospital who resulted positive for SARS-CoV-2 have been categorized according to a 4-level classification: level 0 (asymptomatic, the patient should not be hospitalized), level 1 (mild symptoms, pharyngodynia, dry cough, fever), level 2 (moderate symptoms, high fever, persistent dry cough, asthenia, dyspnea, requires non-invasive oxygen support, may require intensive care), and level 3 (severe symptoms, invasive oxygen therapy, requires access to intensive care). This COVID-19 classification, together with the score of the Sequential Organ Failure Assessment (SOFA), the Charlson Comorbidity Index (CCI) (14), and the other clinical data, was extracted from the electronic case report form for all patients admitted to the ICU between March and April 2020. Biochemical parameters comprised routine coagulation parameters (activated partial thromboplastin activity and ratio, prothrombin activity and international normalized ratio, antithrombin III, thrombocyte count), inflammatory markers (fibrinogen, C-reactive protein, procalcitonin), injury factors (amylase, creatine phosphokinase, lactate dehydrogenase, γ-glutamyl transpeptidase, aspartate aminotransferase, alanine aminotransferase), immune response cells (neutrophils, lymphocytes, monocytes, eosinophils, basophils), proteins (hemoglobin, albumin, creatinine, urea, bilirubin), and minerals (calcium, chloride, phosphorus, magnesium, potassium, sodium). For each parameter, the value at ICU admission, the 72-h mean (the mean in the first 3 days), the 48-h trend (the difference between the value on day 2 and 1), and the 72-h trend (the difference between the value on day 3 and 1) were calculated. The presence of any infection from bronchial aspirates/bronchoalveolar lavage, blood, or urine was arbitrarily coded 1 point, with the co-occurrence of infections in multiple districts being coded as the sum of points (e.g., the presence of a urinary tract infection = 1 point, whereas a urinary tract infection plus lung infection = 2 points).

At baseline, the potential difference between biochemical and respiratory features of patients with hospital-acquired vs. parental-acquired infection was investigated by using paired samples *t*-test for normally distributed continuous values or Mann–Whitney–Wilcoxon (MWW) signed-rank test for skewed continuous values. Skewness was defined by the Shapiro–Wilk test *p* < 0.05, with the 72-h trend of amylase and potassium being the only variables with no normal distribution. Subsequently, the CCI, the body mass index (BMI), the respiratory parameters (PaO_2_/FiO_2_ ratio = P/F; the positive end-expiratory pressure), and all biochemical variables at the 1st day of ICU admission, the 72-h means, and the trends in the first 72 h were associated with the binary clinical outcome (survival:death) in the whole cohort through logistic regression. The length of ICU stay combined with the survival in each outcome group was analyzed through separated linear regressions with the occurrence of infections and with the 72-h mean, the 48-h trend, and the 72-h trend of respiratory parameters. All tests were 2-tailed and performed by using IBM SPSS Statistics 22. The raw data used to support the findings of this study are included within the Supplementary Material as a Microsoft Excel worksheet.

## Results

Thirteen males and five females with severe COVID-19 aged 67.77 ± 9.92 years old were admitted to the ICU of our hospital in March–April 2020 for intensive care support, and 11 of them were deceased for COVID-19-associated complications. At admission, 17 patients were categorized as level 3 COVID-19 severity and were all subjected to invasive oxygen therapy with either oropharyngeal tube or tracheostomy, whereas one patient was classified as an upper level 2 patient since he maintained respiratory autonomy through the helmet. Most patients suffered from co-existing conditions, with five patients being classified as nosocomial infected patients (see Table 1 for details).

**Table 1:** Baseline and outcome data of severe COVID-19 patients admitted to our hospital for intensive care support during the Italian pandemic of 2020.

**Descriptors**	**Severe COVID-19 patients**
	**(*N* = 18)**
**Demographics**	
thnicity	Caucasian
Age (years)	67.77 ± 9.92 (43.66; 81.10)
Gender (male:female)	13:5
Admission (month)	March–April
**Disease severity**	
SOFA	6.67 ± 2.22
CCI	4.06 ± 1.95
Hospital-acquired	5
Parental-acquired	14
Level 0	–
Level 1	–
Level 2	1
Level 3	17
**Clinical outcome**	
ICU stay (days)	17.17 ± 7.18 (7.00; 35.00)
Discharged (*n*)	7
Deceased (*n*)	11

*SOFA, Sequential Organ Failure Assessment; CCI, Charlson Comorbidity Index (scores 1–2 = mild; scores 3–4 = moderate; scores ≥5 = severe), COVID-19 level of severity (level 0 = asymptomatic, the patient should not be hospitalized; level 1 = mild symptoms, pharyngodynia, dry cough, fever; level 2 = moderate symptoms, high fever, persistent dry cough, asthenia, dyspnea, requires non-invasive oxygen support, may require intensive care; level 3 = severe symptoms, invasive oxygen therapy, requires access to intensive care); ICU, intensive care unit*.

Compared with parental-acquired infections, the hospital-acquired infection patients had higher CCI at admission (5.60 vs. 3.46, *t*-test *p* = 0.033), lower baseline positive end-expiratory pressure (11.00 vs. 14.87 cm H_2_O, *t*-test *p* = 0.012), lower 72-h mean of positive end-expiratory pressure (10.87 vs. 14.81 cm H_2_O, *t*-test *p* = 0.014), higher 72-h mean of thromboplastin activity (35.2 vs. 28.46, *t*-test *p* = 0.013), higher 72-h mean of thromboplastin ratio (1.25 vs. 1.02, *t*-test *p* = 0.006), lower antithrombin III at 48-h and 72-h trends (−1.00 vs. 8.77%, *t*-test *p* = 0.005 and −5.40 vs. 9.08%, *t*-test *p* = 0.021), lower baseline fibrinogen (441.40 vs. 647.00 mg/dl, *t*-test *p* = 0.040), lower 72-h trend of amylase (−34.60 vs. −11.00 IU/l, MWW *p* = 0.035), lower hemoglobin at admission and 72-h mean (9.50 vs. 11.75 mg/dl, *t*-test *p* = 0.0004 and 9.26 vs. 11.71 mg/dl, *t*-test *p* = 0.0002), lower 48-h trend of albumin (−0.22 vs. −0.01 g/dl, *t*-test *p* = 0.046), lower potassium at admission and 72-h mean (3.84 vs. 4.44 mmol/l, MWW *p* = 0.009 and 3.93 vs. 4.45 mmol/l, *t*-test *p* = 0.006).

Concerning the survival prediction in the whole cohort, the higher was the baseline BMI (OR 0.437, *p* = 0.031), antithrombin III (OR 0.853, *p* = 0.048), and hemoglobin (OR 0.332, *p* = 0.046), the lower was the risk of death. Conversely, the higher was the polymorbidity represented by a high CCI, the greater was the risk for adverse outcome (OR 2.519, *p* = 0.048). Those patients who survived vs. those who deceased had these baseline predictors of 30.11 ± 3.89 vs. 24.50 ± 2.31 kg/m^2^ for BMI (*t*-test *p* = 0.001), a circulating antithrombin III of 96.71 ± 14.99 vs. 72.73 ± 13.14% (*t*-test *p* = 0.003), hemoglobin of 12.03 ± 1.09 vs. 10.55 ± 1.30 mg/dl (*t*-test *p* = 0.024), and a CCI of 2.71 ± 1.89 vs. 4.91 ± 1.51 (*t*-test *p* = 0.015). No other baseline variables were found to be associated with the survival.

**Figure 1:**
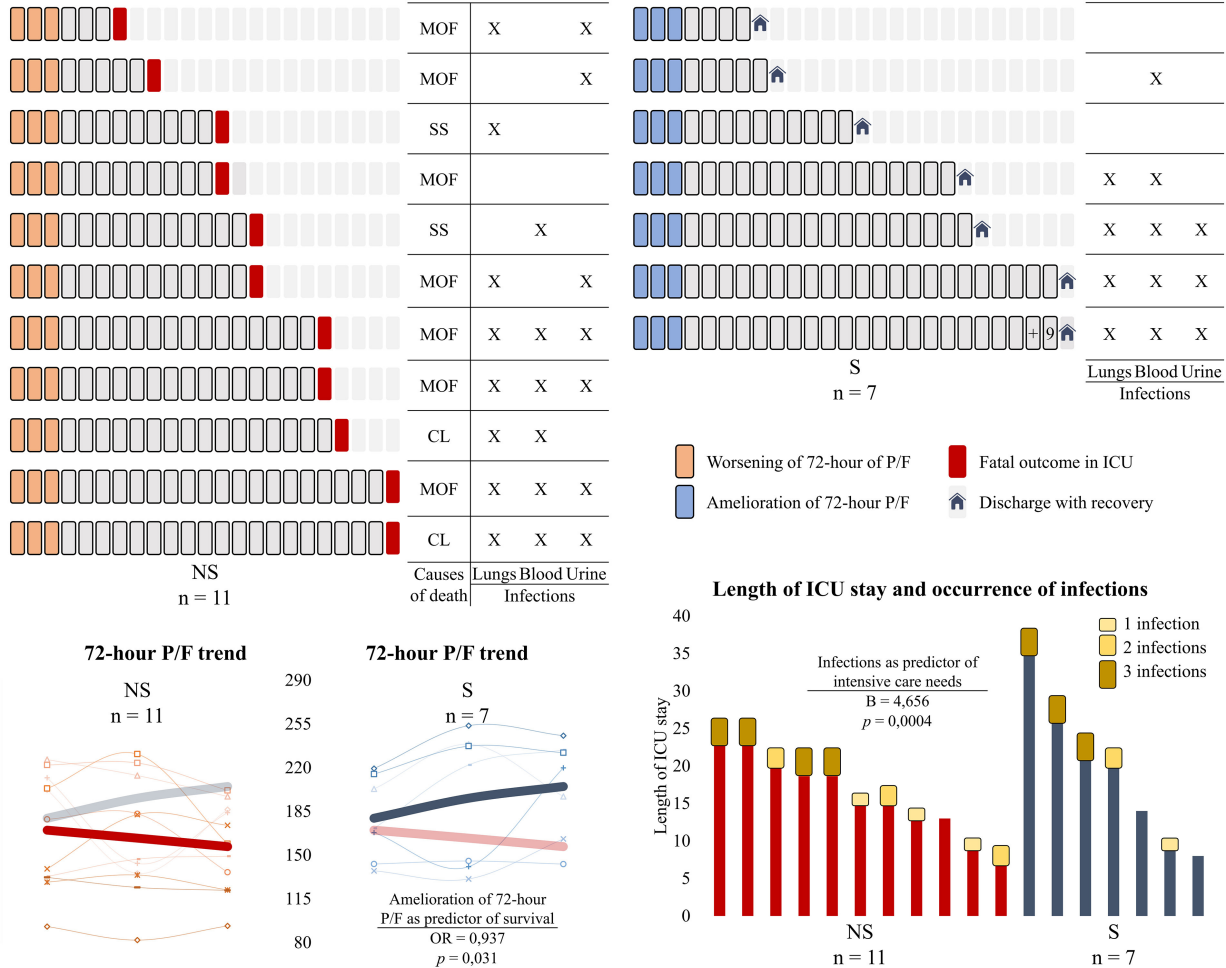
Days of intensive care, survival outcomes, the occurrence of infections, and the 72-h trend of PaO_2_/FiO_2_ in severe COVID-19 patients admitted to the intensive care unit (ICU). Above are represented the length of ICU stay for each patient, with a square corresponding to a day. Patients have been divided into two groups according to the clinical outcome (non-survivors on the left and survivors on the right). The causes of death for the non-survivors are shown in the columns on the left, with the occurring infections being represented for both groups. Below are depicted the line graphs of the 72-h trend of PaO_2_/FiO_2_ ratio and the bar graphs of the days of ICU stay combined with the occurrence of infections. The bold trends in the line graphs represent the average trend of the parameter of interest, with the variable of the other group being represented as nuanced for easier comparison. In our cohort of severe COVID-19 patients admitted to the ICU, the amelioration of the PaO_2_/FiO_2_ in the first 3 days was predictive of survival (OR and *p*-value are shown), whereas the occurrence of infections during the hospitalization was predictive of intensive care needs (B and *p*-value are shown). NS, non-survivors; S, survivors; MOF, multiple organ failure; SS, septic shock; CL, collapsed lungs; PaO_2_/FiO_2_ ratio, P/F.

Considering the 48-h and 72-h trends of the collected variables, only the negative 72-h trend of P/F was found to be predictive of fatal outcome (OR 1.067, *p* = 0.031). Patients who survived had a 72-h trend of P/F of +25.34 ± 25.14 vs. −13.23 ± 24.02 for those who deceased (*t*-test *p* = 0.005). Concerning the length of ICU stay, the non-survivors needed intensive support for 16.00 ± 5.31 days, whereas the survivors stayed in the ICU for 19.00 ± 9.63 days. No respiratory parameters were found to be associated with the days of ICU stay. Conversely, the occurrence of infections resulted to be predictive of the length of ICU stay in the whole cohort (see Figure 1 for details), with B being 4.656 (*p* = 0.0004; 95% CI 2.466:6.846).

## Discussion

We have presented the main clinical and biochemical features of 18 patients with severe COVID-19 that were managed in our ICU during the SARS-CoV-2 pandemic in Italy. The demographic descriptors were in line with the literature evidence, being older males the individuals more prone to both being infected and encountering severe consequences of COVID-19 (15). In addition, we found the highest potential for casualty from nosocomial infections, meaning that the infectious cross-contamination affecting already critical patients, for example, surgical patients, could represent an exponential risk for fatal consequences (16).

The baseline clinical descriptor CCI was found to be associated with ICU death. Undeniably, a common denominator of patients with SARS-CoV-2 is known to be the co-existence of multiple conditions, especially cardiovascular diseases (17). The common underlying conditions in the totality of casualties in Italy have been hypertension in 66.8%, type 2 diabetes in 30.0%, and ischemic heart disease in 27.6% (Italian SARS-CoV-2 Surveillance Group, 25 June 2020). Similarly, our cohort comprised hypertension in 11 (61.11%), type 2 diabetes in 5 (27.8%), and other cardiovascular conditions, such as arrhythmia and vascular diseases, in four cases (22.2%). The BMI, antithrombin III, and hemoglobin at admission resulted to be associated with the risk of death. However, the small sample size of our patients limits the generalization of these parameters beyond the context of our study group. Nevertheless, some evidences in the literature are in line with our observations. A high BMI in older adults is known to be protective against adverse events (18), and the human host is known to face a protracted inflammatory status along with a rapid emptying of body reservoirs during any infective complication (19). As a result, a lower BMI in COVID-19 was associated with ICU mortality in a multiethnic population from the United States (7). Furthermore, decreased hemoglobin in non-survivors is suggested to be associated with SARS-CoV-2 interference with iron metabolism and mimicking of hepcidin roles (20, 21), the latter being an important regulator of oxygen supply (22). Concerning antithrombin III, low plasma concentrations were found in Chinese patients who deceased for COVID-19-associated complications (23). Importantly, the intensive care for our patients comprised equivalent respiration, hydration, nutritional, and pharmacological support with antiviral, anti-inflammatory, antiplatelet, and immunosuppressive drugs, but contrariwise covered a different anticoagulant therapy with low-molecular-weight-heparin (LMWH). The anticoagulant therapy was in fact increased from 4,000 to 6,000 IU of enoxaparin every 12 h after the first literature sheds evidence highlighting the importance of thromboembolic prevention in COVID-19. Antithrombin III is known to be crucial for heparin activity and Xa binding, and low circulating levels are associated with a high risk of thromboembolic events. Still, there is no consensus about the proper pharmacological therapy for COVID-19, but narrow indications support the use of high pharmacological doses of LMWH and treatment with hyperimmune serums (24–26).

The trend of P/F in the first 72 h was a good predictor of clinical outcome, with the negative trend among the non-survivors possibly being ascribed to a late disease stage and therefore to increased alveolar damage. The positive trend in survivors could have also mirrored a good adaptive response to mechanical ventilation and intensive care that hence allowed a discharge with recovery. A significantly better P/F ratio at admission was also observed in patients from the United States who survived in the ICU (7), and its early improvement was associated with discharge with recovery in another Italian cohort (27). Elevated markers of liver injury, high C-reactive protein, and low lymphocytes at admission were associated with adverse outcomes in Chinese patients with COVID-19 (28, 29), but we found no significant changes of the abovementioned parameters possibly associated with medication concealing. The occurrence and the cumulative value of identified co-infections during hospitalization were positively associated with longer ICU stay in both survivors and non-survivors, and this may be linked with the disruption of host defenses that are no longer able to prevent pathogen migrations (30, 31).

Although our patients were relatively young individuals (mean age 67.77 ± 9.92 years), the incidence of mortality of 61.11% was higher than some previous reports, thus certainly contributing to reducing the ICU stay of non-survivors of 3 days less than the average survivors' length of stay. The great case fatality rate could be due to the co-existence of negative prognostic factors, such as polymorbidity, low BMI, and low hemoglobin. Furthermore, both the respiratory features (worsening of 72-h P/F) and the coagulation abnormalities (low antithrombin III) at admission could be indicative of an advanced stage of the disease. More information will be needed to inform intensive care for these challenging patients and therefore characterize both the unpredictable nature of SARS-CoV-2 and the multifaceted features of COVID-19.

## Data Availability Statement

The datasets presented in this study can be found in online repositories. The names of the repository/repositories and accession number(s) can be found in the article/Supplementary Material.

## Ethics Statement

Ethical review and approval was not required for the study on human participants in accordance with the local legislation and institutional requirements. Written informed consent for participation was not required for this study in accordance with the national legislation and the institutional requirements.

## Author Contributions

MB, TC, FP, and MM collected the clinical data and managed the database. MB analyzed the data and wrote the first draft of the manuscript. TC, FP, MM, MP, EDV, GB, and PP revised the first draft and contributed to the manuscript sections. All authors contributed to the manuscript revision and read and approved the submitted version.

## Conflict of Interest

The authors declare that the research was conducted in the absence of any commercial or financial relationships that could be construed as a potential conflict of interest.
